# Deep Transcriptome Sequencing of Two Green Algae, *Chara vulgaris* and *Chlamydomonas reinhardtii*, Provides No Evidence of Organellar RNA Editing

**DOI:** 10.3390/genes8020080

**Published:** 2017-02-20

**Authors:** A. Bruce Cahoon, John A. Nauss, Conner D. Stanley, Ali Qureshi

**Affiliations:** Department of Natural Sciences, University of Virginia’s College at Wise, 1 College Ave., Wise, VA 24293, USA; jan78@pitt.edu (J.A.N.); cds6f@uvawise.edu (C.D.S.); aliq30@gmail.com (A.Q.)

**Keywords:** RNA editing, green algae, *Chara vulgaris*, *Chlamydomonas reinhardtii*, chloroplast, mitochondria

## Abstract

Nearly all land plants post-transcriptionally modify specific nucleotides within RNAs, a process known as RNA editing. This adaptation allows the correction of deleterious mutations within the asexually reproducing and presumably non-recombinant chloroplast and mitochondrial genomes. There are no reports of RNA editing in any of the green algae so this phenomenon is presumed to have originated in embryophytes either after the invasion of land or in the now extinct algal ancestor of all land plants. This was challenged when a recent in silico screen for RNA edit sites based on genomic sequence homology predicted edit sites in the green alga *Chara vulgaris*, a multicellular alga found within the Streptophyta clade and one of the closest extant algal relatives of land plants. In this study, the organelle transcriptomes of *C. vulgaris* and *Chlamydomonas reinhardtii* were deep sequenced for a comprehensive assessment of RNA editing. Initial analyses based solely on sequence comparisons suggested potential edit sites in both species, but subsequent high-resolution melt analysis, RNase H-dependent PCR (rhPCR), and Sanger sequencing of DNA and complementary DNAs (cDNAs) from each of the putative edit sites revealed them to be either single-nucleotide polymorphisms (SNPs) or spurious deep sequencing results. The lack of RNA editing in these two lineages is consistent with the current hypothesis that RNA editing evolved after embryophytes split from its ancestral algal lineage.

## 1. Introduction

Plant organelle RNA editing is a post-transcriptional change in the nucleotide composition of an RNA and in most cases involves a change in a pyrimidine by the addition or removal of an amino group to convert a uracil to or from a cytosine [1]. First discovered in the mitochondria of wheat (*Triticum aestivum*) and evening primrose (*Oenothera berteriana*), this phenomenon appears to repair deleterious mutations and preserve protein function in organelles that are limited to clonal reproduction and prone to a relatively high rate of mutation [2,3,4]. An enzymatic mechanism for these nucleotide changes is not known.

The majority of editing occurs within coding regions [5] and these changes have every possible translational consequence: the correction of an incorrect start codon, repair of an amber mutation, creation of a correct stop codon, conservation of key amino acids, and silent changes that have no effect on amino acid content [6]. RNA editing has also been found outside of transcript coding regions in *Arabidopsis*, tobacco, and *O. berteriana* mitochondria [7,8,9,10]. Some of these edits are necessary for proper intron removal [9,10] but most are difficult to link to a specific change in function and may be superfluous.

Extensive organelle RNA editing occurs among almost all embryophytes [11] with the only known exception being the non-tracheophyte, *Marchantia polymorpha*, which appears to have lost the ability [12,13,14]. There is a general consensus that editing does not occur in green algae and that the phenomenon evolved after the invasion of land or in the extinct algal ancestor of all land plants [15,16]. RNA editing is not exclusive to land plants, however, as it also occurs in peridinin containing dinoflagellates [17]. Dinoflagellates are a diverse collection of unicellular algae grouped within the Alveolata superphylum of the SAR protist supergroup [18] whose plastids do not share a common lineage but were independently obtained from red algae, green algae, haptophytes, and diatoms between 50 and 200 million years ago [19,20,21]. Extensive plastid RNA editing has been found in *Ceratium horridum*, *Heterocapsa triquetra*, *Lingulodinium polyedrum*, and *Symbiodinium minutum* [17,22,23,24], all of which occur in a single branch of dinoflagellates that contain the peridinin pigment, suggesting these have plastids of red algal origin.

In addition to the sequence-derived evidence for RNA editing, in silico methods have been developed that use evolutionarily conserved mRNA edit sites among embryophytes to predict sites in newly produced genomic sequences [25,26,27,28,29,30]. One of these, PREPACT 2.0, predicts two edit sites in the green alga *Chara vulgaris* [29] which belongs to the Charophyceae, a sister clade to the embryophytes [31].

The lack of published evidence for the absence of RNA editing in green algae, the presence of editing in two photosynthetic lineages, the in silico prediction of editing in *Chara*, plus the potential for easily overlooked non-coding region edits led us to undergo a complete survey of organelle transcriptomes of two green algae, *C. vulgaris* and *Chlamydomonas reinhardtii*, chosen to represent the Streptophyta and Chlorophyta algal lineages [32]. Initial screening suggested putative edit sites existed within both species but subsequent analyses and experiments including Sanger sequencing, high resolution melt analysis, and RNase H-dependent PCR (rhPCR) determined the transcripts were not edited.

## 2. Materials and Methods

### 2.1. Strains and Culturing

*C. vulgaris* was dredged from a pond on the UVa-Wise campus (Wise, VA, USA), March 2015, and a culture was initiated in a 20 liter aquarium along with mud and water from the same pond. The culture was maintained in the UVa-Wise greenhouse with natural light. The tank water was replenished periodically with pond and/or distilled water.

*C. reinhardtii*, strain cc503, was obtained from the *Chlamydomonas* stock center (St. Paul, MN, USA; www.chlamycollection.org), January 2015, and maintained on Tris–acetate–phosphate (TAP) medium with Hutner’s trace elements [33]. Liquid cultures for DNA and RNA extraction were grown to mid-log phase in bubble cultures at 25 °C and illuminated at 500 μE m^−2^ s^−1^ using a combination of LED and plant growth lights.

### 2.2. Deep Sequencing and Genome Assembly

*C. vulgaris* thalli were cut from the greenhouse grown culture, washed multiple times in distilled water to remove contaminating algae and bacteria, and excess water was blotted from them with paper towels. Thalli were immediately placed in liquid nitrogen and ground into a powder using a mortar and pestle. DNA or RNA was extracted using Qiagen’s DNeasy and RNeasy kits (Valencia, CA, USA), according to the manufacturer’s instructions, and RNA extraction included the optional DNase treatment. Nucleic acid yields were determined using a Nanodrop Lite (Thermo Scientific, Waltham, MA, USA) apparatus. *C. reinhardtii* cells were pelleted in a clinical centrifuge at 2000× *g* for 5 min, the growth medium was decanted, and the pellet was immediately resuspended in extraction buffer.

*Chara* samples used for deep sequencing were collected in April and July 2015. Illumina sequencing of the *Chara* genome and April transcriptome sample libraries was completed by the University of Virginia’s sequencing core (Charlottesville, VA, USA). Illumina sequencing of the July *Chara* and *C. reinhardtii* total transcriptome libraries was completed by Genewiz (South Plainfield, NJ, USA).

Plastome and chondriome sequences were assembled for *Chara* using GENEious software v. 8 [34] and GenBank archived sequences as references (plastome: NC_008097; chondriome: NC_005255) [35,36].

### 2.3. RNA Editing Detection

Each transcriptome was aligned to chondriome and plastome genomic sequences and putative edit sites were initially determined using GENEious’ “Find Variations/SNPs” function set at a minimum variant frequency of 0.1. This very low editing frequency threshold was chosen to minimize the possibility of overlooking edits due to a low editing frequency. This was based on the deep transcriptome alignment and analysis of edit sites found in the whole chondriome of *Nicotiana tabacum* [7] where a significant portion of the edited transcripts occurred as a relatively low percentage of the total sequenced transcripts (Figure S1). The same threshold (0.1) would have found 96.4% of tobacco edits. All sites in *Chara* and *C. reinhardtii* with at least 10% of the transcripts having a discrepancy from the genomic reference sequence were then manually inspected to determine if any differences were caused by phenomena other than RNA editing.

### 2.4. High Resolution Melt Analysis of Chara psbI

High resolution melt (HRM) has been used previously to define edit sites [37] and uses the melt-curve function of a quantitative PCR (qPCR) apparatus to determine the temperature at which a PCR generated amplicon denatures. It detects nucleotide composition between two nucleic acids by the difference in their melt temperatures.

One gram of *Chara* was removed from the greenhouse culture, rinsed with distilled water, and placed in 100 ml of liquid medium containing ¼× TAP salts and ¼× Hutner’s trace elements [33] in 250 mL beakers sealed with parafilm. The pH was adjusted to 6, 7, or 8 using phosphate buffers. *Chara* was incubated in triplicate in each temperature + pH regimen for 3 days in a temperature controlled growth chamber on a 16 h light/8 h dark cycle and 200 μE m^−2^ s^−1^ of light.

Thalli were harvested from the growth medium, blotted dry, immediately placed in liquid nitrogen, and ground into a powder using a mortar and pestle. DNA and RNA were extracted separately using Qiagen’s DNeasy and RNeasy kits, according to the manufacturer’s instructions, and RNA extraction included the optional DNase treatment. Nucleic acid yields were determined using a Nanodrop Lite apparatus. Complementary DNA (cDNA) was produced from DNase treated RNA using Bio-Rad’s iScript cDNA synthesis kit (Hercules, CA, USA).

Amplicons were produced as paired PCR reactions, one genomic DNA (gDNA) for each cDNA, harvested from the nine pH + temperature conditions using Bio-Rad’s Precision Melt Supermix with 25 ng of gDNA or cDNA template and 10 pmol of each primer. Primers were designed with the aid of Primer3 [38]: psbI-F 5′-AGAATTTGAATTCGAATATT, psbI-R 5′-TGTTTGTATATACAGTTGTA, psbA-F 5′-AATTAGTACCATGGCTTTTA, psbA-R 5′-AAGTTATGAGCATTACGTTC. Amplicons were produced with the program (95 °C 3 min, [95 °C 30 s, 52.7 °C 30 s, 72 °C 30 s] ×39). Melt temperatures were determined from *psbI* (experimental) and *psbA* (negative control) using the melt analysis protocol of Bio-Rad’s CFX96 Real-Time PCR system programmed to heat from 65 °C to 95 °C at 0.1 °C increments. Data were collected from gDNA and RNA extracted from three *Chara* replicates and three technical replicates for a total of *n* = 9 melt temperature differences for each environmental condition. Melt temperature differences were calculated by subtracting the lower melt temperature from the higher one. Two-way analysis of variance (ANOVA) of the melt temperature differences was completed using SigmaStat v. 4.0 (Systat Software, San Jose, CA, USA).

### 2.5. RNase H-Dependent PCR Analysis of Chara psbI

rhPCR is a test that can distinguish single nucleotide polymorphisms (SNPs) between two otherwise identical template DNAs using qPCR [39]. rhPCR primers were designed to anneal to the putative edit sites of *Chara*’s *psbI* gene according to the criteria and technical help provided by Integrated DNA Technologies (Coralville, IA, USA), psbI-rh-F 5′-TTACGTCCTGGATTACGTCuGGGAA-x psbI-rh-R 5′-GTATATACAGTTGTAATATTgTTTAA-x (Figure S2). The -x represents a 3′ blocked nucleotide that suppresses initiation of DNA polymerization. The lowercase nucleotide represents a ribonucleotide within the DNA primer that is cleaved by RNase H2 in the event of a non-Watson–Crick base pairing. This removes the blocked 3′ nucleotide and allows extension from the primer.

Prior to experimental data collection, a range of RNase H2 from 2 mU to 1 nU was tested to find the optimal concentration of enzyme for each primer pair. Primer dilutions from 20 pM to 1 pM were also tested. Final reaction components for *Chara psbI* reactions included 25 nM DNA or cDNA template, 10 pmol of each primer, PerfeCTa SYBR green supermix (Quanta-bio, Beverly, MA, USA), 0.01% Triton X-100, and 8 nU RNase H2 (Integrated DNA Technologies). qPCR was performed in Bio-Rad’s cfx96 Real-Time PCR system with the program (95 °C 3 min, [95 °C 10 s, 52.7 °C 5 s, 60 °C 30 s] ×45). A rhPCR *N. tabacum* positive control was designed based on an edit site in the mitochondrial cytochrome oxidase b (*cob*) gene (Genbank BA000042, nucleotide 40,964) using the primers 5′-TTACTATTGGACAAATTTCTuCTTA-x and 5′-TCTGGGACGAGTTGGAAGAG and amplified using the same strategy described above (Figure S3).

### 2.6. Sanger Sequencing

*psbI* genomic fragments from *Chara* were PCR amplified using the primers 5′-AGTGGTCGAAAGCGACAGAT and 5′-TTGTTTGGCAAGCATCAGTT. *psbI* mRNA from *Chara* grown at 20 °C, 25 °C, and 30 °C was converted to cDNA using Bio-Rad′s iScript cDNA synthesis kit and PCR amplified using the same primers. Control reactions pre-treated with RNase prior to cDNA synthesis were also conducted to ensure that the amplicons were produced from cDNA and not DNA contaminating the template RNA. The genomic *cob* region of *C. reinhardtii* was PCR amplified with the primers 5′-ATCTCGTTACCACCCAACCA and 5′- TTCTTGGAACGGTGGTTCTC sequenced. All amplicons were directly sequenced using Sanger methodology by Genewiz with the same primers used to produce the amplicons.

## 3. Results

### 3.1. C. vulgaris Organellar Genomes

Since SNPs and RNA editing would be indistinguishable in our assay, it was necessary to sequence the plastome and chondriome of the *C. vulgaris* culture established for this study. Using a template based assembly strategy, 728,947 reads aligned to the 67,737 base pair (bp) chondriome and 1,523,432 reads aligned to the 184,933 bp plastome. Compared to the GenBank archived sequences, the assembled plastome differed by 21 SNPs—nineteen in intergenic regions and introns, one synonymous change in *rps3*, and one Gly115Val missense in *rpl22* (Table 1). The chondriome differed by six SNPs within the two *rns* (small ribosome subunit) gene exons. The newly generated plastome and chondriome sequences were used as templates for transcriptome alignment and analysis.

### 3.2. C. vulgaris Transcriptome RNA Editing Screen

To find potential edit sites, total RNA was extracted from *C. vulgaris* thalli in April (20 °C) and July 2015 (30 °C) and sequenced as separate replicates with yields of 43,741,464 paired-end reads for the April extraction and 187,760,146 for July.

In the mitochondrial transcriptome, 67,949 reads from the April replicate and 12,418,978 reads from the July replicate aligned to the chondriome. One hundred and seventy nucleotide variants were found and investigated. All of them occurred outside of protein coding regions and upon manual inspection, each was found to be the result of misaligned sequences or due to introns directly adjacent to intron/exon junctions. None of them were likely candidates for RNA editing.

**Figure 1:**
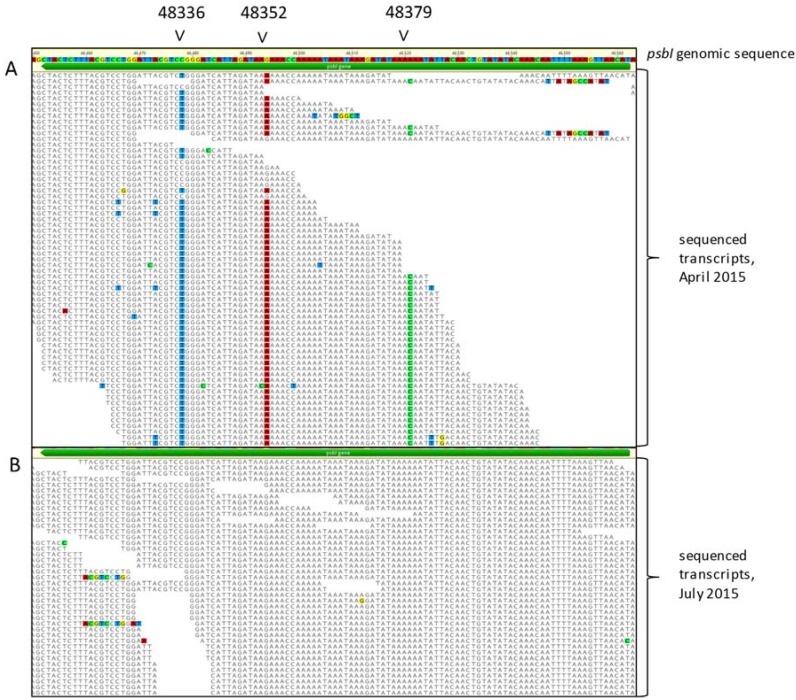
Alignment of deep sequenced transcriptome fragments to the *Chara vulgaris* chloroplast genome. (**A**) The chloroplast transcriptome of *C. vulgaris* collected from a greenhouse grown culture in April 2015. The mean coverage was 50.5 ± 22.9 with a min:max of 2:79. Three putative RNA edit sites were detected at bases 48,336, 48,352, and 48,379. (**B**) The transcriptome was re-sequenced from RNA extracted from tissue collected from the same culture in July 2015. The mean coverage was 5860.5 ± 619.7 with a min:max of 3,622:6,496. The three putative edit sites were not detected in this sample.

In the plastid transcriptome 1,530,450 reads aligned to the plastid genome from the April replicate, 32,533,027 from the July replicate, and 637 nucleotide variants were identified. Thirty-seven occurred within protein coding regions and were manually inspected. Thirty-four were misaligned transcript sequences, but three potential synonymous edit sites occurred within the *psbI* coding region from the April replicate. These variants did not appear in the July library (Figure 1). This raised two possibilities: (1) The library prepared from one of the RNA sample misamplified a transcript with several errors or (2) RNA editing is occurring in *psbI* and the editing process is environmentally regulated. To test the environmental regulation hypothesis, *Chara* was harvested from the greenhouse culture and incubated at pH 6, 7, and 8 and 20 °C, 25 °C, and 30 °C. Temperature was considered a viable option due to the difference between the ambient greenhouse temperature at the times of the two collections, and RNA editing has been shown to respond to temperature in *Drosophila melanogaster* [40]. pH was chosen as a variable after noticing that the greenhouse culture had become progressively more alkaline since its establishment.

**Figure 2:**
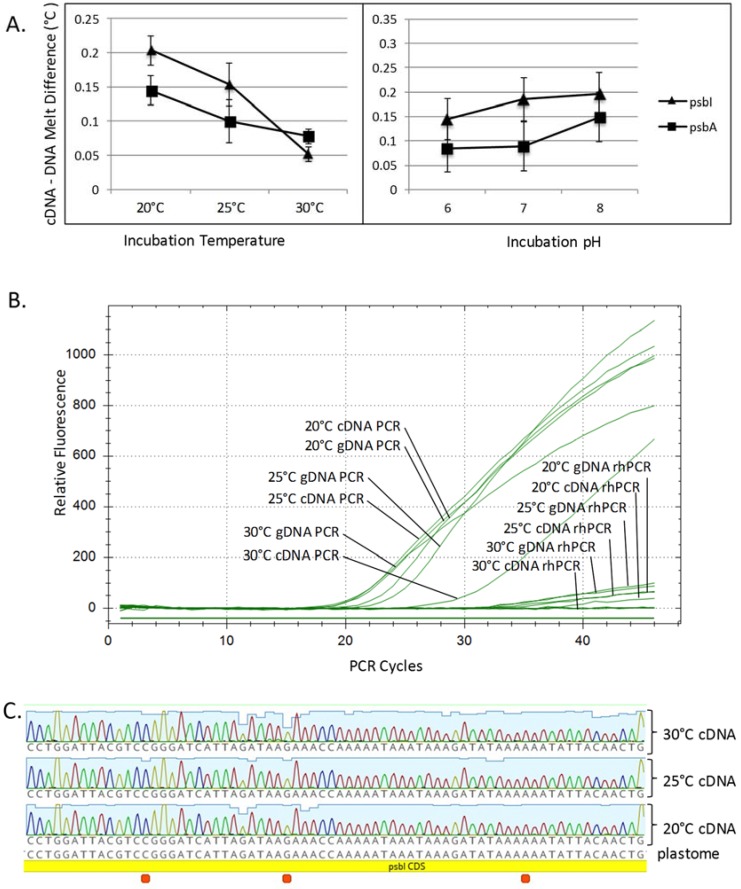
Three follow-up experiments attempting to detect three putative RNA editing sites in the *C. vulgaris psbI* mRNA. (**A**) *Chara* was grown in nine different environmental conditions: 20 °C, 25 °C, 30 °C and pH 6, 7, 8, in triplicate, and then DNA + RNA were extracted from each. The melt temperatures for genomic DNA (gDNA) and complementary DNA (cDNA) were determined as paired reactions using high resolution melt analysis. Melt temperature differences between gDNA and cDNA derived amplicons were calculated by subtraction. Data presented on these graphs are the averages of nine observations ± standard error (SE). There were qualitative differences in the cDNA–DNA melt temperatures of *psbI* and the negative control, *psbA*, at the different incubation temperatures but these differences were statistically insignificant. Different pH also had no significant effect on the cDNA–DNA melt temperatures and two-way analysis of variance detected no interaction between the temperature and pH for either gene; (**B**) RNase H-dependent PCR (rhPCR) was used in an attempt to detect single base changes between the *psbI* gDNA and cDNA extracted from *Chara* incubated in different temperatures. This assay was designed to suppress PCR amplification of the genomic sequence but not cDNA made from an edited transcript. The quantitative PCR (qPCR) amplification curves show that gDNA and cDNA amplified with sigmoidal curves with standard PCR but that both gDNA and cDNA were suppressed by the addition of an rhPCR primer despite the presence of RNase H2 in the reactions. This suggests no editing occurred at any of the tested temperatures; (**C**) cDNAs were PCR amplified and sequenced from total RNA extracted from *Chara* grown at 20 °C, 25 °C, and 30 °C. The sequences all matched the genomic sequence, suggesting no editing had taken place.

HRM analysis was used to measure nucleotide changes between *psbI* genomic DNA and the putative edited mRNA in *Chara* grown under the different temperature and pH conditions. Melting temperatures were determined for genomic and cDNA amplicons from two genes, *psbI* with three putative edit sites and a portion of *psbA* with no edit sites. There was an intriguing qualitative difference between the cDNA and gDNA melting points of *psbI* grown at different temperatures (Figure 2A), but two-way ANOVA analysis determined these were insignificant (*p* = 0.135). There were no significant changes noted in *psbI* or *psbA* when the pH of the growth medium differed and no significant interaction between temperature and pH.

Deep sequencing and HRM had produced conflicting or statistically questionable results so another technique, rhPCR, was used to determine if RNA editing was creating differences between *psbI* DNA and mRNA [39]. The rhPCR assay allows the detection of SNPs by differentially suppressing the synthesis of amplicons that differ from one another by a single base; further description can be found in the Materials and Methods and Figure S3. The rhPCR assay was designed to suppress PCR amplification from the gDNA template but not a cDNA made from an edited mRNA. There are no references describing the use of rhPCR to detect RNA edit sites, so this technique was tested using a previously characterized RNA edit site in the tobacco *cob* gene (Figure S3). For the *Chara psbI*, the putative edit sites were close enough that both forward and reverse primers could be designed to suppress non-edited gDNA (Figure S2). qPCR assays were run with paired forward and reverse rhPCR primers or with a single rh primer paired with a standard one. No matter the combination of primers, there was nearly equal suppression of rh primer extension from both the cDNA and gDNA templates from *Chara* grown at either 20 °C, 25 °C, or 30 °C (Figure 2B), suggesting that no editing had occurred.

Finally, *psbI* transcripts from *Chara* grown at 20 °C, 25 °C, and 30 °C were reverse transcribed and sequenced using Sanger methodology. No differences were found at the putative edit sites between transcripts extracted from *Chara* grown at differing temperatures (Figure 2C) and there were no overlapping peaks within the electropherograms as would be expected with a mixture of edited versus non-edited transcripts.

### 3.3. C. reinhardtii Transcriptome RNA Editing Screen

To screen for potential editing in *C. reinhardtii*, ribosome depleted total RNA from strain cc503 was sequenced and aligned to the Genbank archived chloroplast (FJ423446.1) and mitochondrial (EU306622.1) sequences for this strain. For the chloroplast, 32,533,027 RNA derived reads aligned to the 184,933 bp plastome and for the mitochondria, 12,418,978 reads aligned to the 67,737 bp chondriome.

**Figure 3:**
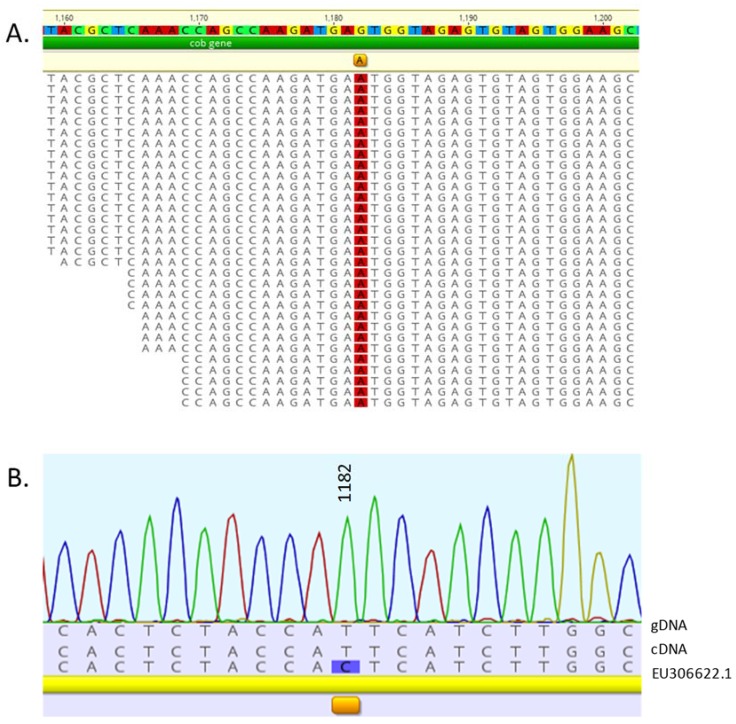
Alignment of the deep sequenced mitochondrial transcriptome of *Chlamydomonas reinhardtii* strain cc503. (**A**) A single putative RNA editing site was detected in the cytochrome oxidase b gene at nucleotide 1,182. The mean coverage of the *cob* gene was 28,371.5 ± 7,598 with a min:max of 816:42,177. (**B**) Sequencing of the *cob* DNA and cDNA from the cc503 isolate used for the deep sequencing revealed a SNP at that site, therefore no editing had taken place.

Using the same methodology described for *Chara*, one putative edit site was detected in the mitochondrial nucleotide position, 1182, within the cytochrome oxidase b coding region (Figure 3A). The *cob* gene region from the cc503 isolate used for the transcriptome analysis was sequenced and found to be identical to the mRNA Illumina reads, not the archived genomic sequence (Figure 3B), therefore this nucleotide change was due to a SNP, not RNA editing. No edit sites were detected in the chloroplast.

## 4. Discussion

Plant cell organelles, plastids and mitochondria, carry their own reduced chromosomes and have gene expression machineries reminiscent of their prokaryotic ancestry. They differ from bacterial systems, however, with numerous unique adaptations that are likely a direct result of their endosymbiotic origin. One striking aspect of plant organelle gene expression is the expanded role of RNA processing, including endonucleolytic cleavage of polycistronic transcripts, 5′ and 3′ end maturation, and RNA nucleotide editing [41]. These RNA processing mechanisms rely upon an extensive array of nuclear encoded pentatricopeptide repeat proteins that comprise a significant proportion of the thousands of proteins that must be imported into these organelles from the cytoplasm [42,43]. The focus of this study, RNA editing, is found among nearly all land plants but has never been reported in green algae. Although there is a broad assumption that this does not occur among algae, to the best of our knowledge there are no published reports of these negative results. In this study, an exhaustive screen for RNA editing was performed on two green algae and both were negative. These negative results impact two hypotheses regarding this phenomenon.

**Hypothesis** **1.** *RNA editing in embryophytes originated either after the invasion of land or in the now extinct algal ancestor of all land plants*.

Extensive RNA editing occurs among almost all embryophytes suggesting it has a monophyletic origin [11]. Among tracheophytes, all appear to edit organellar transcripts with no known exceptions. Among non-tracheophyte land plants, RNA editing has been found to occur in the hornwort *Anthoceros formosae* [44], and in the moss *Physcomitrella patens* [45,46], but is missing in the liverwort *Marchantia polymorpha*, which appears to have once had the ability to edit but lost it after its radiation from other bryophytes [12,13,14].

The origin of RNA editing may have occurred after the invasion of land or in a now extinct algal ancestor of land plants. This was tested by sequencing the genome and transcriptome of *C. vulgaris*, a member of the charophyte clade of Streptophyte algae which contain the genera most closely related to land plants [32]. This, along with a recent in silico prediction of two edit sites in the *Chara* plastome [30], led us to screen the organellar transcriptomes of this species for the presence of RNA editing. Deep sequencing of these transcriptomes followed by rigorous testing of putative editing sites found during this screen suggests that organelle RNA editing does not occur within this species and that this process must not have existed in the common ancestor of land plants and Charophytes.

**Hypothesis** **2.** *RNA editing convergently evolved in land plants and dinoflagellates*.

Extensive organellar RNA editing does not occur among green algae but it has been found in another protist lineage, peridinin-containing dinoflagellates. The processes of plastid RNA editing found in dinoflagellates and tracheophytes differ from one another in extent and range. RNA editing among the dinoflagellates is extensive, ranging from 0.31% in *H. triquetra* to 7.17% in *Karenia mikimotoi* [22,47] whereas plant plastid mRNAs are edited at an average frequency of 0.1% [42]. The range of nucleotides edited in dinoflagellates is also more extensive with every conceivable nucleotide transition occurring amongst the species that have been analyzed to date [19], whereas in higher plants the nucleotide transition is mostly C–U. These differences along with the large evolutionary distance between these two groups has lead several groups to propose that RNA editing arose independently in these two lineages [20,24].

Dinoflagellates are secondary and tertiary endosymbionts and would be related to algae primarily through the organelles that the dinoflagellate host cells acquired. Considering the occurrence of RNA editing in two very different groups of photosynthetic organisms (land plants and dinoflagellates) and the possibility that *C. vulgaris* may have lost the ability to edit, similar to *Marchantia*, we expanded the screen to include another green alga representing a different lineage, the well-studied model organism *C. reinhardtii*. No evidence of RNA editing was found. This fails to reject the hypothesis that land plants and dinoflagellates convergently evolved RNA editing abilities.

## 5. Conclusions

It is assumed that organellar RNA editing does not occur in algae, but there are no published accounts offering empirical evidence of its absence and a recently published in silico screen of the *C. vulgaris* plastome predicted there may be edit sites within coding regions. In this study, the transcriptomes of two green algae were deep sequenced and thoroughly screened for organellar editing. Some putative edit sites were initially detected but subsequent experiments determined these were either spuriously detected, or a single nucleotide polymorphic difference between the clonal isolate that was sequenced versus the archived genome sequence. Based on these results we can say with confidence that there is no organellar RNA editing in these two species.

## Figures and Tables

**Table 1:** Single nucleotide polymorphisms (SNP) found between the plastomes and chondriomes of *Chara vulgaris* thalli collected for this study in Wise, VA, USA and the Genbank archived sequences from thalli collected in Québec City, QC, Canada [35,36].

*Chara vulgaris* chondriome SNPs compared to NC_005255			
**SNP location**	**nt change**	**gene**	
65107	G → C	*rns*	
65109	T → G	*rns*	
65189	C → T	*rns*	
66731	G → C	*rns*	
66768	T → C	*rns*	
66802	A → C	*rns*	
***Chara vulgaris* plastome SNPs compared to NC_008097**			
**SNP location**	**nt change**	**gene**	**effect**
7191	T → A	intergenic	
24408	G → T	intergenic	
37112	G → T	*ycf3* intron 1	
42310	T → A	intergenic	
44130	C → G	intergenic	
44734	G → A	intergenic	
44967	T → C	intergenic	
61795	C → T	*trnK*(uuu) intron 1	
87704	C → G	intergenic	
92194	G → T	intergenic	
94397	T → G	intergenic	
100725	G → A	intergenic	
107118	G → A	intergenic	
115615	A → C	*petB* intron 1	
126737	A → T	intergenic	
129523	G → A	*rps3*	synonymous
129668	C → A	*rpl22*	G115V
137046	T → G	intergenic	
156998	G → C	intergenic	
157889	G → C	intergenic	
183703	A → C	intergenic	

## Supplementary Materials

The following are available online at www.mdpi.com/2073-4425/8/2/80/s1, Figure S1: Distribution of tobacco chondriome edit sites based on the number of edited transcripts at each site compared to the total, Figure S2: *Chara vulgaris* rhPCR primer annealing locations, Figure S3: rhPCR detection of edited mRNA using the *Nicotiana tabacum cob* gene.
