# Supplementary material for: Deep Transcriptome Sequencing of Two Green Algae, Chara vulgaris and Chlamydomonas reinhardtii, Provides No Evidence of Organellar RNA Editing
Source: Genes (Basel). 2017 Feb 20;8(2):80. doi: 10.3390/genes8020080 (PMC5333069; doi:10.3390/genes8020080)
Supplement: Supplementary file 1 [file genes-08-00080-s001.docx]

Supplementary Materials: Deep Transcriptome Sequencing of Two Green Algae, *Chara vulgaris* and *Chlamydomonas reinhardtii*, Provides No Evidence of Organellar RNA Editing

**A. Bruce Cahoon, John A. Nauss, Conner D. Stanley and Ali Qureshi**

**Figure S1.** Distribution of tobacco chondriome edit sites based on the number of edited transcripts at each site compared to the total. Graphs were made by re-analyzing data from Grimes et al. (2014). (**A**) Box plot representation of all edit sites with descriptive statistics. (**B**) The number of edit sites found with transcript edit frequencies within 10% intervals. The majority of edits (365 out of 635) found within the tobacco chondriome had an RNA editing frequency over 90%. Twenty-three were below 10%.

**Figure S2.** *Chara vulgaris* rhPCR primer annealing locations.

**Figure S3.** rhPCR detection of edited mRNA using the *Nicotiana tabacum cob* gene. (**A**) The portion of the tobacco cytochrome oxidase gene used as a positive control for rhPCR. A previously defined edit site, nt 40,964 of GenBank BA000042, is denoted by a yellow oval with an ‘E’, and primer annealing sites are denoted by green pointed boxes. The rhPCR primer on the left (rh_Nt_cob_fwd) anneals to the edited site with a single ribonucleotide, the remainder of the primer is made of deoxynucleotides, and the 3′ deoxynucleotide is modified to block extension by DNA polymerase. This primer was designed such that if it annealed to an unedited gDNA, the template extension by DNA polymerase would be suppressed. If it annealed to a cDNA made from an edited mRNA, the enzyme RNase H2 will cleave at the RNA:DNA mismatch, the modified 3′ nucleotide will be removed, and extension can proceed. (**B**) qPCR curves showing *cob* gDNA and cDNA standard PCR amplification compared to rhPCR. When standard PCR was performed using Nt_cob_fwd and Nt_cob_rev primers, sigmoidal amplification curves were produced with crossover thresholds of 14 and 18 cycles. When the forward primer was replaced by the rhPCR primer, PCR amplification of gDNA and cDNA was severely suppressed. The addition of RNase H2 relieved the suppression when the template was edited cDNA but had no effect on non-edited gDNA.
